# Size-controlled gold nanoparticles on octahedral anatase particles as efficient plasmonic photocatalyst

**DOI:** 10.1016/j.apcatb.2017.01.043

**Published:** 2017-06-05

**Authors:** Zhishun Wei, Lorenzo Rosa, Kunlei Wang, Maya Endo, Saulius Juodkazis, Bunsho Ohtani, Ewa Kowalska

**Affiliations:** a Institute for Catalysis, Hokkaido University, N21 W10, 001-0021 Sapporo, Japan; b Centre for Micro-Photonics, Swinburne University of Technology, PO Box 218, Hawthorn, 3122 Australia; c Department of Information Engineering, University of Parma, V.le G.P. Usberti 181/A, I-43124 Parma, Italy

**Keywords:** Gold nanoparticles, Octahedral anatase particles, Plasmonic photocatalysts, 3D-FDTD simulations, Titania

## Abstract

Octahedral anatase particles (OAPs), prepared by ultrasonication-hydrothermal reaction (US-HT), were modified with 2 wt% of gold by photodeposition. Conditions of US-HT process such as durations of US and durations of HT were varied to obtain OAPs products different by physicochemical and morphological properties. Au/OAPs samples were characterized by X-ray diffraction (XRD), scanning transmission electron microscopy (STEM), X-ray photoelectron spectroscopy (XPS) and diffuse reflectance spectroscopy (DRS). The photocatalytic activity was tested under UV irradiation for decomposition of acetic acid (CO_2_ system) and dehydrogenation of methanol (H_2_ system) under aerobic and anaerobic conditions, respectively, and for oxidation of 2-propanol under visible light irradiation. Photodeposition of gold was very fast for all OAPs samples (0.5–10 min) under Ar atmosphere, and the clear correlation between the content of electron traps (ETs) and the induction period, during which nanoparticles (NPs) of gold are formed, indicates that ETs in titania samples are a key-factor for rapidity of gold photodeposition on titania surface. It was found that better morphology of titania (larger content of faceted particles) resulted in formation of larger gold NPs, while small gold NPs were deposited on structural defects. Modification of OAPs with gold NPs resulted in significant enhancement of photocatalytic activity, being e.g., 1.5 (CO_2_ system), 7.7 (H_2_ system), and even more than 40 under vis irradiation. It was found that both the properties of titania and gold are crucial for resultant photocatalytic activity, but a direct correlation between one structural/physical property and photocatalytic activity could not be obtained since all structural properties changed simultaneously when conditions of photocatalyst preparation (US-HT) were changed. Therefore, gold NPs of controlled sizes were deposited on OAPs product with the best morphology by modified photodeposition method. Clear correlation between photocatalytic activity under visible light and the size of gold NPs indicates that gold properties are decisive for visible light activity rather than titania properties. 3D-FDTD simulations confirm that an increase in the size of gold NPs results in extended surface areas with field enhancement.

## Highlights

• Gold-modified octahedral anatase particles (Au/OAPs) with enhanced photocatalytic activity.
• Structural/lattice defects as nucleation sites for gold NPs.
• Size-controllable gold NPs by photodeposition method in the presence of various hole and electron scavengers.
• Enhanced vis-photocatalytic activity with an increase in gold NPs’ size.
• Strong enhancement of plasmon resonance for larger gold NPs by 3D-FDTD simulations.

## Graphical abstract

**Figure:**
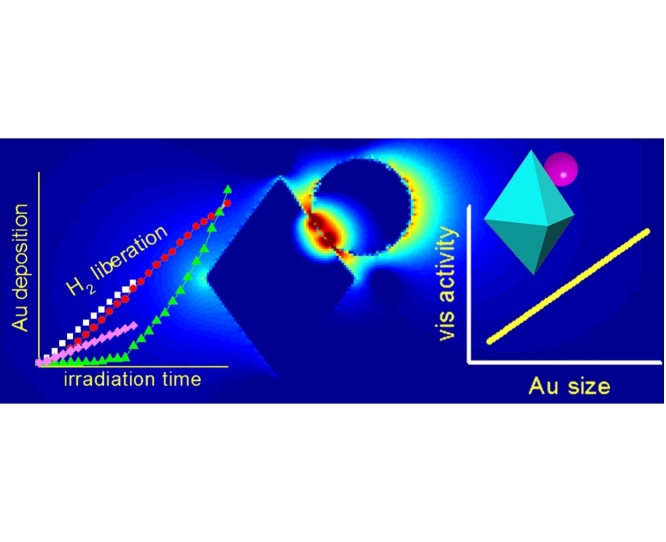

## 1 Introduction

Semiconductor photocatalysis has been extensively studied for more than forty years for environmental purification [1], [2], [3], anti-fogging/self-cleaning surfaces [4], water splitting [5] and solar energy conversion [6]. It is expected that solar photocatalysts (semiconductor photocatalysis driven by the Sun) could help in solving the most emerging humanity problems, i.e., (1) energy, (2) water and (3) environment (three of “Top Ten Problems of Humanity” proposed by Prof. Smalley [7]), where abundant and clean energy would enable the resolution of all other problems. Solar energy conversion into electricity and fuel (e.g., H_2_, CH_3_OH) by novel photocatalytic systems has recently been extensively studied, e.g., inspired by nature, two-step photoexcitation systems, the so-called “artificial photosynthesis” [8].

Titanium(IV) oxide (titania) is one of the most often used semiconductor photocatalysts because of its high photoreactivity, chemical stability, high availability and non-toxicity (except possible nanomaterial toxicity [9]) [10]. However, two shortcomings for its application should be overcome, i.e., recombination of charge carriers (e^−^/h^+^, typical for all semiconducting materials) and inactivity in the visible range of the solar spectrum (due to its wide band-gap, ca. 3.2 eV depending on polymorphic form). Therefore, plenty of studies have been performed to improve the performance of titania photocatalysts by morphology arrangements [11], [12], surface modification [13], [14], doping [15], [16], [17], and coupling with other semiconductors [18]. Noble metals in the form of adsorbed complexes [19], [20], [21] and metallic deposits [22], [23], [24], [25], [26], [27] have been extensively investigated for improvement of photocatalytic activity of titania under UV irradiation [28], [29]. Enhancement of photocatalytic activity comes from prolongation of lifetime of charge carriers [25] since noble metals serve as electron sinks [30], and thus accelerate the transfer of electrons from titania to substrates, e.g., protons to evolve hydrogen [31], [32].

Over the last decade another property of noble metals (Au, Ag), i.e., absorption of visible light due to plasmon resonance, has been used to activate titania [33], [34] and other wide band-gap semiconductors (CeO_2_ [35], [36], ZnO [37]) towards visible light. Plasmonic photocatalysts (photocatalysts which use plasmonic properties of noble metals) have been considered as a good candidate for efficient conversion of solar energy to chemical energy (solar fuel) and/or electrical energy (by solar cells), and purification of environment [34], [38], [39], [40], [41]. Although plasmonic properties of noble metals were observed 100 years ago, explained more than 30 years ago and commercially used in many fields (biosensors [42], chemical sensors [43], [44], nano-lithography/nano-photonics [45], [46], surface enhanced Raman spectroscopy (SERS) [42], [44], medicine (drug delivery, cancer therapy) [44] and optical data storage [47]), the examination of their usage for photocatalysis was started ca. ten years ago [33]. Despite the novelty of plasmonic photocatalysis, a large number of studies have already been performed to improve photoactivity and stability as well as to clarify the mechanism under irradiation with visible light, i.e., energy transfer, electron transfer, plasmonic heating [48], [49], [50]. A few reviews on plasmonic photocatalysis have also been published [38], [39], [51], [52], [53].

The large majority of research has been performed using commercial titania nanoparticles (NPs) as a support for plasmonic NPs (mainly P25 composed of anatase and rutile crystallites and amorphous titania [54]), which makes discussion on plasmonic photocatalysis quite complex, e.g., due to impurities, irregular morphology, and possible transfer of charges between phases [55]. Our recent study indicates that faceted titania of octahedron shape (octahedral anatase particles, OAPs) of high-level photocatalytic activity (due to preferential distribution of shallow than deep electron traps resulting in high mobility of electrons [11]) is a good support for NPs of noble metal such as gold, silver and copper [56]. It has been found that gold-modified titania showed the best photocatalytic activity under visible light irradiation among tested metals. Therefore, OAPs have been selected as a support for gold NPs in the present study. It has been expected that preparation conditions of titania, resulting in the change of surface properties (crystalline size, specific surface area, crystallinity) and content of OAPs in the final product (morphology) [11], [57], as well as method of gold deposition should influence the final properties of gold NPs (size, shape and distribution on OAPs), and thus resultant photocatalytic activity. Accordingly, the influence of titania morphology and properties of gold deposits on photocatalytic activity was investigated and is presented in this paper.

## 2 Experimental details

The detailed procedure for synthesis of OAPs has been reported elsewhere [11], [58]. In brief, OAPs were prepared by hydrothermal reaction (HT, at 433 K for 3–24 h) of titanate nanowires (TNWs). Prior to HT, ultrasonication (US, 0–4 h) was used to homogenize TNWs suspension as well as to influence the morphology of TNWs (by their shortening) and thus to change the morphology of the final product (content of OAPs). Gold was photodeposited on OAP samples (prepared at various US-HT conditions) under UV/vis irradiation (λ > 290 nm) in the presence of methanol (MeOH) as a hole scavenger and under argon (Ar) atmosphere to avoid scavenging of photogenerated electrons by oxygen (O_2_) [59]. In brief, five hundred mg of an OAP product was used for each photodeposition, and the amount of gold was calculated to be 2 wt% of titania. The weighed OAP powder was put into a Pyrex glass tube equipped with a magnetic stirrer, to which 25 mL of MeOH (50 vol%, MiliQ water) was added. Then, the aqueous solution of chloroauric acid was slowly dropped while being stirred. The suspension was Ar-sparged for 15 min. The tube was sealed with a rubber septum and photoirradiated with magnetic stirring (500 rpm) by a 400-W high-pressure mercury lamp (Eiko-sha) under thermostatic control at 298 K (details presented elsewhere [60]). The codes of gold-modified OAPs samples were defined according to conditions of US-HT process, e.g., 1US/1HT means gold-modified sample, in which OAPs were obtained for 1-h US and 1-h HT at 433 K (in total nine different OAP samples were modified with gold). After photodeposition, samples were washed (three times with methanol and then three times with Milli-Q water), centrifuged and freeze dried.

To control solely the size of gold NPs (keeping the properties of titania the same), modified photodeposition method was used for OAP sample of the best morphology, i.e., prepared for 1 h of US and at 433 K of HT for 6 h [11]. Three modified photodeposition systems were used: (1) without Ar prebubbling (initial aerobic conditions): MeOH/air sample and (2) for O_2_-saturated MeOH suspension (15-min O_2_ prebubbling): MeOH/O_2_ sample; in those two systems (MeOH/air and MeOH/O_2_) photogenerated electrons were firstly consumed by O_2_ hindering formation of gold NPs, and (3) in the presence of 2-propanol (IPA; 50 vol%) as a hole scavenger (instead of methanol) under Ar atmosphere: IPA/Ar sample. The gold-modified sample (1US/6HT) prepared at standard conditions (under Ar from deaerated MeOH suspension) was named as MeOH/Ar (for easier comparison between those four samples).

Photocatalysts were characterized by X-ray diffraction (XRD), scanning transmission electron microscopy (STEM, HITACHI HD-2000), X-ray photoelectron spectroscopy (XPS) and diffuse reflectance spectroscopy (DRS). XRD analysis was performed using the Rigaku intelligent X-ray diffraction SmartLab system equipped with a sealed tube X-ray generator (a Cu target). Crystallite sizes of anatase and gold were estimated from the corrected width of an anatase 101 and gold 200 diffraction peaks using the Scherrer equation. Crystallinity was estimated using highly crystalline nickel oxide as an internal standard, and experimental details were presented in previous paper [57]. XPS analysis was conducted on a JEOL JPC-9010MC (MgKα X-ray) spectrometer. DRS measurements were performed on a JASCO V-670 spectrophotometer equipped with a PIN-757 integrating sphere. Barium sulfate and respective bare OAP products were used as references.

Finite difference time domain (FDTD) method was used to calculate light-field distributions and extinction spectra (total extinction losses, due to light absorption and scattering). For this purpose, the structure was surrounded by a total-field scattered-field broadband source, extending from 200 nm to 1500 nm wavelength, and two monitor boxes recording the total extinction cross-section and the scattering cross-section of the structure. Another monitor recorded the E-field intensity distribution in the total-field domain to give the field maps showing the hotspot distribution around the geometry.

Photocatalytic activities were tested under UV–vis irradiation for oxidative decomposition of acetic acid (CO_2_ system) and anaerobic dehydrogenation of methanol (H_2_ system). In each experiment, 50 mg of the photocatalyst was suspended in 5 mL of aqueous solution containing 5 vol% acetic acid (CO_2_ system) or 50 vol% methanol (H_2_ system), sealed with a rubber septum, and then photoirradiated with magnetic stirring (1000 rpm) under air (CO_2_ system) or argon (H_2_ system). Photoirradiation was carried out in the same irradiation set-up as that used for gold photodeposition. Amounts of liberated CO_2_ and H_2_, respectively, were determined in gas phase by gas chromatography (TCD-GC). The visible light activity for oxidation of 2-proponol was evaluated by determination of the amount of acetone generated. Fifty mg of the photocatalyst was suspended in 5 mL of 2-propanol (5 vol%) and irradiated under magnetic stirring in a photoreactor equipped with a 300-W xenon lamp (CX-04E Inotech, Japan), cold mirror, water filter and cut-off filter (Y45 (λ > 420 nm), Asahi Techno Glass). Dark reactions in a testing tube (the same as used for photoirradiation experiments) thoroughly covered with aluminum foil were also carried out. The photoreactor was described in detail and shown in previous reports [59], [60]. The generated acetone was analyzed in liquid phase, after sample filtration through a syringeless filter (Whatman Mini-UniPrep, PVDF), by gas chromatography (Shimadzu GC-14B, equipped with a flame ionization detector).

## 3 Results and discussion

### 3.1 Gold-modified OAPs samples (OAPs with different physicochemical and surface properties)

#### 3.1.1 Preparation of Au/OAPs

Gold was photodeposited on OAPs samples with different surface properties, i.e., prepared at various conditions of US-HT process. In total, nine OAPs samples, prepared at 433 K of HT, were used as gold supports, where duration of either HT or US was changed from the standard conditions (standard conditions: 1 h of US and 6 h of HT; at which the OAPs sample of the best morphology was prepared [11]). The resultant gold-modified samples were named according to the conditions of US-HT process, as the following: 1US/3HT, 1US/4.5HT, 1US/6HT (prepared at standard conditions of US-HT process), 1US/12HT, 1US/24HT, 0US/6HT, 2US/6HT, 3US/6HT and 4US/6HT. The properties of OAPs samples are shown in Table 1.

**Table 1:** Properties of bare OAPs samples (prepared at various conditions of US-HT process) and crystallite sizes of gold NPs; SSA-specific surface area.

code	US time/h	HT time/h	Crystallinity (%)(a)	size/ nm(a)	SSA/ m^2^ g^−1^(a)	total OAP content (%)(a)	*d*_ETs_/ mmol g^−1^(a)	Au size/nm
1US/3HT	1	3	28	13	250	7	0.239	2.6
1US/4.5HT	1	4.5	60	16	170	35	0.158	3.9
1US/6HT	1	6	78	17	124	64	0.114	5.2
1US/12HT	1	12	83	19	81	61	0.098	6.4
1US/24HT	1	24	81	19	80	62	0.065	6.5
0US/6HT	0	6	80	17	131	51	0.118	4.7
2US/6HT	2	6	80	17	130	53	0.108	5.6
3US/6HT	3	6	76	17	122	50	0.113	5.0
4US/6HT	4	6	78	17	129	45	0.111	5.1

(a) Values measured and reported previously [11].

**Fig. 1:**
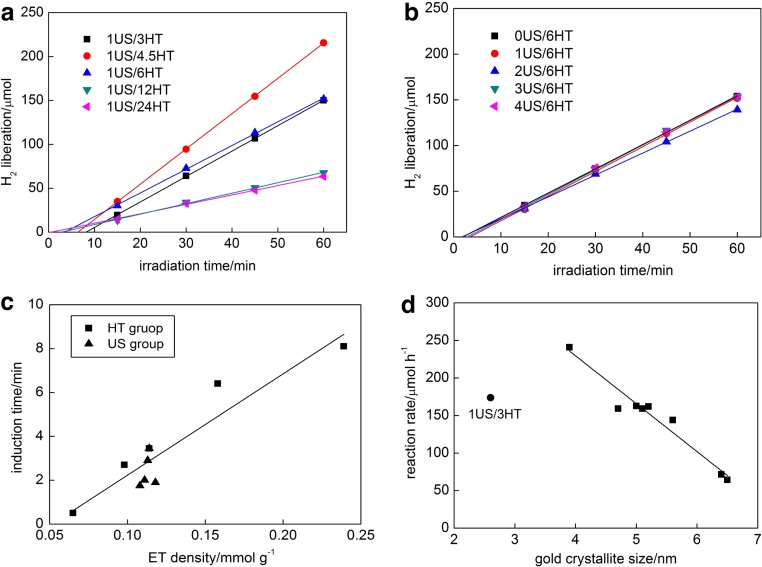
Hydrogen evolution during gold deposition on OAPs samples prepared with: (a) various durations of HT reaction and constant duration of US (1 h) and (b) various duration of US and constant duration of HT reaction (6 h); (c) The correlation between the induction period of hydrogen evolution and content of ETs (HT group: change in duration of HT reaction; US group: change in duration of US); (d) The correlation between crystallite size of gold and reaction rate of hydrogen evolution.

The course of hydrogen evolution during gold deposition on OAPs samples is shown in Fig. 1 . The induction period (intersection with the x-axis), during which NPs of gold are formed, depends on the properties of titania (e.g., specific surface area, crystallinity, crystallite size and the content of electron traps (ETs)). For example, undetectable induction period was observed for gold photodeposition on well crystalized titania with large NPs of rutile (TIO5 from Catalysis Society of Japan) [61], ca. 2-min induction period for titania with fine anatase NPs (ST01 from Ishihara) [62], [63], and ca. 4-min one for gold deposition on titania with fine rutile NPs with large content of ETs (TIO6 from Catalysis Society of Japan with 17-fold larger content of ETs than that in TIO5 sample: 242 μmol g^−1^ *v.* 14 μmol g^−1^ of Ti^+3^ [64]) [61]. Present study on OAPs confirms that content of ETs in titania sample is a key-factor for rapidity of gold photodeposition on titania surface, as shown in Fig. 1(a) and Table 1. The shortest induction period was obtained for 1US/24HT sample, prepared at longest duration of HT, with the smallest content of ETs (65 μmol g^−1^ [11]). The clear correlation between the induction period and the content of ETs was obtained, as shown in Fig. 1(c). In contrast, OAPs samples prepared with various durations of US do not significantly differ in their properties (except in morphology) and thus the induction periods in all of those samples were almost the same (1.5–3.5 min), as shown in Fig. 1(b). Only 2US/6HT sample showed slightly shorter induction period of 1.8 min, which clearly correlated with the smallest content of ETs (108 μmol g^−1^) for that sample.

**Fig. 2:**
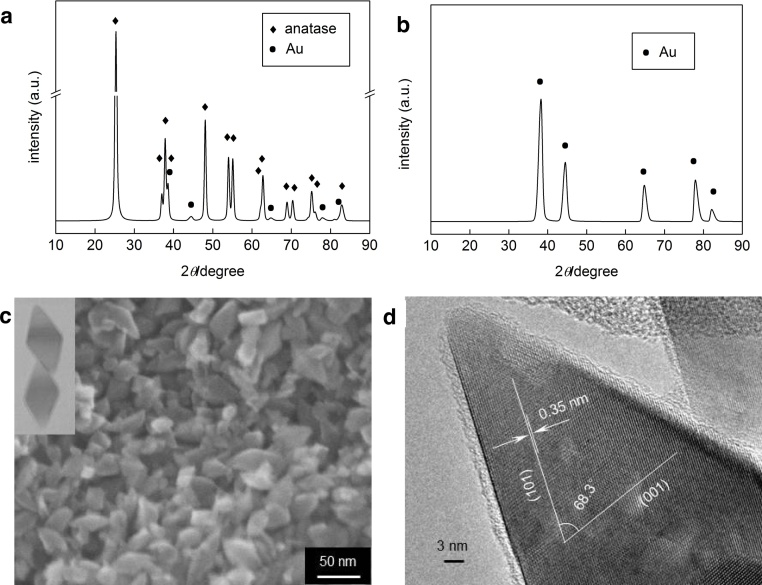
(a–b) XRD pattern of 1US/6HT sample: (a) original pattern (◆ –− anatase titania, ● – gold) and (b) gold pattern – after subtraction of anatase peaks; (c-d) Microscopic images of bare OAPs prepared with 1-h US and 6-h HT: (c) SEM image and (d) HRTEM image.

**Fig. 3:**
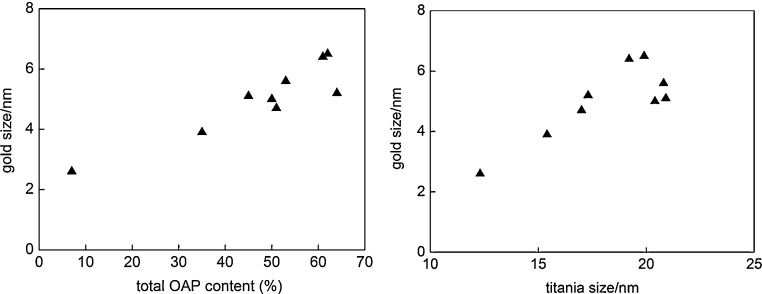
(left) Influence of titania morphology (OAP content) and (right) Influence of crystallite size of titania on the crystallite size of gold NPs.

**Fig. 4:**
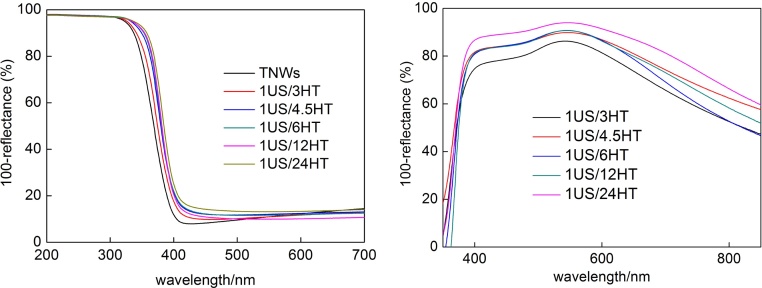
DRS of (left) TNWs and bare OAPs (BaSO_4_ as reference) and (right) Au/OAPs samples (with bare OAPs samples used as a reference).

Interestingly, the induction periods do not correlate with the rate of hydrogen evolution, which is quite surprising since usually the fastest metal deposition (gold and other noble metals) means also the highest rate of hydrogen evolution, i.e., the easier reduction of metal cation during photodeposition is, the larger is the reduction power for hydrogen evolution [56], [61], [63]. It should be pointed that in the present system, OAPs are formed from TNWs, which have semiconducting properties and similar band-gap (ca. 3.2 eV, Fig. 4(left)) to titania. Therefore, shorter duration of HT reaction means larger possibility of incomplete conversion of TNWs into OAPs. The study on OAPs indicated that 3-h and 4.5-h HT reaction resulted in incomplete conversion of TNWs into anatase [11], [58]. It was proposed that 6-h HT reaction at 433 K was sufficient for efficient TNWs conversion, and this product possessed the best morphology (content of particles with octahedron shape) and photocatalytic activity among all other OAPs samples [11]. Therefore, it should be remembered that the OAPs products prepared at 3 h and 4.5 h of HT reaction contain also TNWs. Accordingly, gold was deposited on the surface of both TNWs and OAPs. The highest rate of hydrogen evolution for 1US/4.5HT sample (containing TNWs and OAPs) indicates some synergetic effect of both nanostructures (reaction rate of hydrogen evolution during gold photodeposition on TNWs <100 μmol h^−1^), which will be investigated in detail in our future study. At present, it is proposed that the presence of TNWs caused the generation of smaller sizes of gold NPs (Table 1, i.e., ca. half smaller crystallite size of gold in 1US/3HT and 1US/4.5HT samples than in others), which is not surprising since gold NPs are mainly formed on crystal defects [34], [65], and TNWs as mainly amorphous phase possess large amount of defects. For reaction of hydrogen evolution, the uniform distribution of metal deposits on all semiconductor NPs and large specific surface area are crucial for efficiency. Therefore, small NPs of gold uniformly deposited on TNWs/OAPs, large specific surface area (due to TNWs) and sufficient crystallinity (due to OAPs) result in enhanced evolution of hydrogen. Similarly to data of induction periods, OAPs samples prepared with various durations of US (with similar surface properties, except in morphology) exhibit almost the same photocatalytic activity for hydrogen evolution (ca. 160 μmol h^−1^). Only, 2US/6HT sample showed slightly lower rate of hydrogen evolution (ca. 144 μmol h^−1^), due to smaller gold-titania interface as a result of the largest crystallite sizes of gold (5.6 nm) resulting from the smallest content of ETs (108 μmol g^−1^) in that sample. Interestingly, the good correlation between crystallite size of gold and reaction of hydrogen evolution was found, as shown in Fig. 1(d), with the only one exception for 1US/3HT sample. The exception for that sample (much lower activity than expected based on the gold size) is not surprising since conversion of TNWs into OAPs was very low for 3-h HT reaction (only 7% of OAPs) and thus low crystallinity (28%) and large content of ETs (239 μmol g^−1^) resulted in fast recombination of charge carriers.

#### 3.1.2 Characterization and photocatalytic activity of Au/OAPs

XRD measurements were used for characterization of crystallinity, crystalline composition and crystallite sizes. Summarized data have been already shown (Table 1) and partly discussed in the previous section. Gold was detected in all samples as shown in exemplary XRD pattern of 1US/6HT samples in Fig. 2(a–b). Fig. 2(c–d) shows representative SEM and TEM images of bare OAPs prepared with 1-h US and 6-h HT. Many octahedral-shape NPs are easily observed in the SEM images, and the HRTEM image (Fig. 2d) with the fringes corresponding to (001) and (101) lattice spacing and an angle of 68.3° between them confirms anatase form of titania, as already reported [66].

It was found that crystallite size of gold depended on morphology and crystallite size of titania, i.e., the larger was the content of OAPs in the final product and the larger were the sizes of titania, the larger gold NPs were (Fig. 3). Min et al. proposed that gold NPs were preferentially formed on lattice defects [65]. Similarly, our previous study for commercial titania samples showed the dependence of crystallite size of gold NPs on crystallite size of titania particles, where small anatase with larger number of surface defects than that in well crystallized large particles of rutile [67] stimulated formation of fine gold NPs [34]. It is proposed that the “deep” ETs (attributable to surface defects) in OAPs rather than “shallow” ETs (attributable to surface-reconstruction structure [58]) are active sites for the nucleation of gold NPs (OAPs samples prepared at various durations of US have almost the same content of ETs). Titania of nonperfect crystal structure (large content of surface defects and/or TNWs) stimulated formation of large number of fine gold NPs, while growth of gold NPs happened on perfectly shaped OAPs.

Au-OAPs samples exhibited dark violet color due to photoabsorption by gold NPs (localized surface-plasmon resonance, LSPR), suggesting the presence of spherical gold NPs [59]. Diffuse reflectance spectra of all samples did not differ significantly with λ_max_ of LSPR at ca. 545 nm (Fig. 4). Only slight stronger scattering at longer wavelengths (800–1200 nm) for samples prepared for longer HT reaction (1US/24HT, 1US/12HT) confirms the presence of gold NPs with larger sizes in those samples [63].

The surface composition and chemical states of elements estimated by XPS are shown in Table 2, Table 3. For samples prepared for 1-h US and with various durations of HT reaction, the ratio of oxygen to titanium slightly exceeded the ratio of chemical formula of titania (TiO_2_; O:Ti = 2), reaching 2.3–2.4 for samples prepared with shorter (3–4.5 h) HT reaction, and ratio of 3.0–3.7 for longer (6–24 h) HT reaction. The enrichment of the titania surface with oxygen has been recently reported [56] as a result of extension of HT reaction and thus the larger content of hydroxyl groups adsorbed on titania surface. The larger content of hydroxyl groups in the case of OAPs samples prepared for longer HT reaction was proved by deconvolution of oxygen peak, as shown in Table 3. The highest content of lattice oxygen was detected for OAPs samples prepared with short HT reaction, i.e., ca. 60% (3–4.5 h HT) v. 40–48% (6–24 h HT). In contrast, for OAPs samples prepared for 6-h HT and with various durations of US, the O:Ti ratio was almost constant reaching 1.8–2.0, with the exception of 1US/6HT sample with the ratio of 3.3. Slightly lower O:Ti ratio than 2.0 resulted from substitution of surface oxygen by gold or the decrease in the adsorbed water on the titania surface. For example, it was reported that modification of titania (ST01 from Ishihara) with 2 wt% of gold caused a decrease in O:Ti ratio from 6.9 to 2.7 [63], and modification of OAPs (slightly different than that shown here) with 0.5 wt% of gold resulted in a decrease from 2.5 to 2.0 [56].

**Table 2:** Surface composition of Au/OAPs samples and fraction of oxidation states of Au determined by XPS analysis.

Samples	Content (at.%)				Ratio		Au content (wt.%)	Au 4f_7/2_ (%)		
	Ti	O	C	Au	O/Ti	C/Ti		Au(δ+)	Au(0)	Au(δ-)
1US/3HT	18.6	43.1	38.1	0.22	2.3	2.0	2.91	1.1	91.6	7.3
1US/4.5HT	18.1	43.7	37.6	0.24	2.4	2.1	3.27	0.0	94.0	6.1
1US/6HT	9.5	31.6	58.7	0.17	3.3	6.2	4.41	2.1	93.2	4.7
1US/12HT	9.6	35.4	54.8	0.20	3.7	5.7	5.15	3.7	90.3	6.0
1US/24HT	11.9	36.3	51.5	0.24	3.0	4.3	4.95	2.0	93.5	4.5
0US/6HT	25.8	47.9	26.0	0.38	1.9	1.0	3.63	2.0	96.7	1.3
2US/6HT	27.6	50.5	21.4	0.50	1.8	0.8	4.47	4.5	95.5	0.0
3US/6HT	21.7	44.1	33.9	0.35	2.0	1.6	3.97	3.6	96.4	0.0
4US/6HT	22.8	44.1	32.7	0.39	1.9	1.4	4.22	1.2	98.8	0.0

**Table 3:** Fraction of oxidation states of Ti, O and C from deconvolution of XPS peaks of Ti 2p_3/2_, O 1 s and C 1s.

Samples	Ti 2p_3/2_ (%)		O 1 s (%)			C 1 s (%)		
	Ti^4+^	Ti^3+^	TiO_2_	Ti-OH^a^	Ti-OH^b^	C—C	C—OH	C=O
1US/3HT	96.3	3.7	62.7	18.2	19.1	73.0	17.1	9.9
1US/4.5HT	98.4	1.6	60.1	28.9	10.9	75.2	15.6	9.2
1US/6HT	97.5	2.5	46.9	34.5	18.6	73.5	16.8	9.7
1US/12HT	97.8	2.2	40.5	12.3	47.2	67.5	20.7	11.8
1US/24HT	99.4	0.6	47.7	17.4	34.8	66.2	24.0	9.8
0US/6HT	97.6	2.4	78.5	17.6	3.9	75.4	13.8	10.8
2US/6HT	98.2	1.8	82.8	14.4	2.7	75.1	15.2	9.7
3US/6HT	98.8	1.2	73.3	21.4	5.3	72.9	16.7	10.3
4US/6HT	98.1	1.9	74.9	19.8	5.2	76.2	14.4	9.4

Ti-OH^a^: Ti-(OH)-Ti/Ti_2_O_3_/C=O, Ti-OH^b^: Ti-OH/C—OH.

The form of carbon did not significantly differ in all gold-modified OAPs samples. Carbon 1 s region was deconvoluted for three peaks at binding energies (BE) of ca. 284.8 eV, 286.1 eV and 288.6 eV, which could be assigned to C—C, C—OH and C=O bonds, respectively, and obtained contents were 66.2–76.2%, 13.8–24.0% and 9.4–11.8%, respectively. Carbon originates from the atmosphere during preparation of samples for XPS measurements and is always detected in titania samples [68]. Titanium in all gold-modified OAPs samples existed mainly in Ti^4+^ form (96.3–99.4%). The extension of the duration of HT treatment resulted in the formation of a more crystalline TiO_2_ structure with a smaller amount of oxygen vacancies, i.e., Ti^3+^ (3.7%–0.6% with increase of HT duration from 3 h to 24 h), while the change in duration of US did not significantly influence the titanium state (detailed data shown in Table 3).

**Fig. 5:**
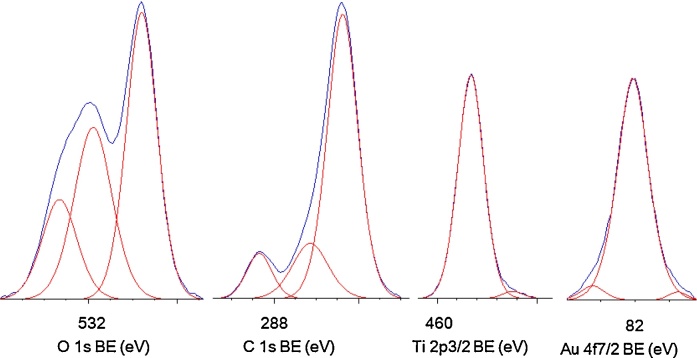
Exemplary XPS results for 1US/6HT sample after deconvolution of O 1s, C 1s, Ti 2p_3/2_ and Au 4f_7/2_ peaks.

The presence of gold was confirmed in all modified samples, as shown in Table 2 and Fig. 5. The content of gold exceeded the amount used for deposition (2 wt%) reaching ca. 2.9–5.15, which was expected (and previously reported [69]), since gold was deposited on the titania surface. Surprisingly, the content of gold directly increases with an increase in the crystallite size of gold estimated by XRD (Fig. S1 (left)). Usually, opposite behavior is observed, ie., smaller sizes of metallic deposits occupied larger area of titania surface, and thus the content of metal on titania surface is higher, e.g., a decrease in the crystallite size of gold NPs from 11.7 nm to 5.1 nm resulted in an increase in the surface content of gold from 2.5 wt% to 4.7 wt% [56]. Present data could be explained by significant differences in specific surface area between samples, especially those prepared at short HT reaction, and thus possessing large content of unreacted TNWs, e.g., 250 m^2^ g^−1^ and 80 m^2^ g^−1^ for 1US/3HT and 1US/24HT, respectively. Considering those differences, the clear correlation between the crystallite size of gold NPs and the ratio of specific surface area to gold content could be obtained (Fig. S1 (right)). Deconvolution of Au peak [70] confirmed that gold deposition resulted in the formation of mainly zero-charged Au (90–99%), as shown in Table 2.

**Fig. 6:**
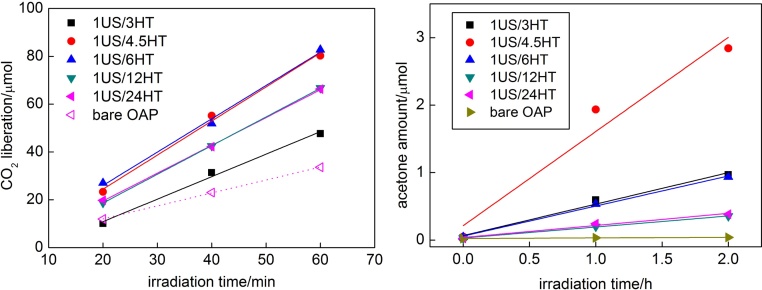
Photocatalytic activity of Au/OAPs samples: (left) under UV/vis irradiation, and (right) under vis irradiation (activity of bare OAPs prepared with 1-h US and 24-h HT was shown for comparison).

The photocatalytic activity of Au/OAPs samples was tested for oxidative decomposition of acetic acid under UV/vis irradiation and oxidation of 2-propanol under vis irradiation, and the data obtained are shown in Fig. 6. Modification of OAPs with gold resulted in significant enhancement of photocatalytic activity for all Au/OAPs samples, reaching even double value of reaction rates for 1US/4.5HT, 1US/12HT and 1US/24HT samples (Fig. 6(left)). However, the clear correlation between the properties (both for titania and gold) and UV-photocatalytic activity could not be obtained. Therefore, it should be concluded that the balance between sufficient specific surface area, sufficient crystallinity, small crystallite sizes of both titania and gold, and morphology results in the best photocatalytic performance under UV irradiation.

**Fig. 7:**
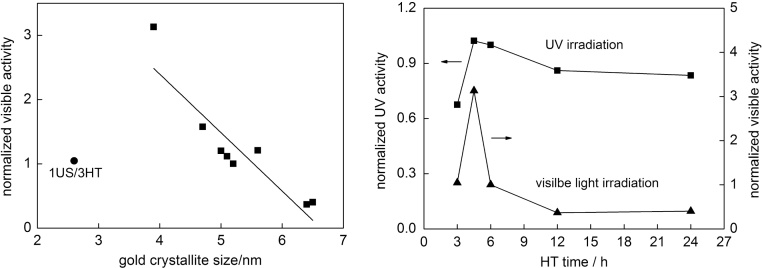
(left) The correlation between crystallite size of gold NPs and vis-photocatalytic activity; (right) The influence of duration of HT reaction on the photocatalytic activity of Au/OAPs samples (Data are normalized to that of photocatalytic activity of 1US/6HT sample).

**Fig. 8:**
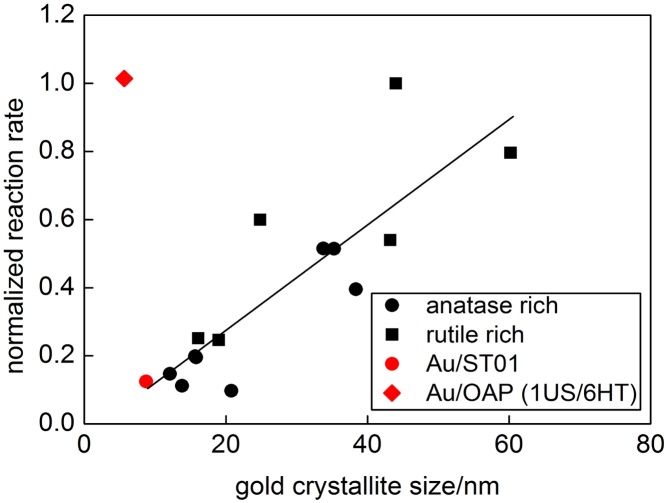
Dependence between crystallite size of gold and photocatalytic activity for commercial (data reported previously [34]) and octahedral titania (1US/6HT).

The highest photocatalytic activity under visible light irradiation was obtained for 1US/4.5HT sample, as shown in Fig. 6 (right). It should be pointed that for that sample (with one of the smallest gold NPs of 3.9 nm) catalytic (dark) activity of gold could not be neglected, as observed by nonlinear evolution of acetone (R^2^ = 0.9607; faster at the beginning of irradiation). Indeed dark activity of 1US/4.5HT sample reached ca. 23% of the overall activity. Catalytic activity in the dark was reported for commercial titania photocatalysts modified with gold by photodeposition, but the levels of those activity were much lower than that during irradiation, and the content of dark activity in the overall activity increased with a decrease in gold size [59]. In contrast to UV-photocatalytic activity, quite clear correlations between photocatalytic activity and properties could be drawn for oxidation of 2-propanol under vis irradiation, as shown in Fig. 7(left) and Fig. S2. For example, the larger specific surface area and the larger content of gold on the surface result in enhancement of photocatalytic activity (Fig. S2), with the exeption of 1US/3HT sample (similar to photocatalytic activity for H_2_ evolution (Fig. 1d) due to large content of TNWs in that sample). Surprisingly, a decrease in crystallite size of gold NPs resulted in an increase in photocatalytic activity, which is opposite to our previous data for various commercial (15) and self-synthesized titania samples (e.g., Fig. 8) [34], [70]. Previously, it was concluded that an increase in crystallite size of gold meant also higher polydispersity of gold NPs, and thus broader LSPR, which directly correlated with photoabsorption of more photons and therefore with an increase in the overall vis activity [59]. At present, an increase in photocatalytic activity with a decrease in gold size could be explained by an increase of gold-titania interface. However, it should be reminded that in those samples a direct correlation between one structural/physical property and photocatalytic activity cannot be obtained since all structural properties change simultaneously when conditions of photocatalyst preparation and/or treatment were changed.

Preparation of different sizes and shapes of gold deposits for enhancement of photocatalytic activity has been extensively investigated and contradictory results have been published. For example, reduction in gold size results in increase in photocatalytic activity for oxidation of alcohols [55], acetone [71] and oxalic acid (e.g., decrease in gold NPs size from 18 nm to 5 nm resulted in two times increase in activity) [72]. In contrast, an increase in gold size resulted in enhancement of decomposition of Plasmocorinth B dye [73], hydrogen evolution during dehydrogenation of isopropyl alcohol [74], photocurrent generation [75], phenol oxidation [70], 2-propanol oxidation [34], and reduction of 2,2′-dipyridyl disulfide and nitrobenzene [76]. Existence of optimal size of gold crystallites depending on support properties was also reported, e.g., 5.3-nm on titania P25 and 7.7-nm on anatase [77]. The gold loading also significantly influenced the resultant photocatalytic activity, and similarly to gold particle size, contradictory results could be found in the literature, i.e., increase of photoactivity with an increase [78] and a decrease [71], [79] in the amount of deposited gold. The existence of optimal gold amount (2–3 wt% [55] and 0.1 wt% [78]) or optimal thickness of gold film (15-nm) [77] was also proposed. It is important to notice that other properties could change with an increase in gold amount, such as particle size, interface between gold and support, light absorption properties (“inner filter” effect and/or light scattering), and thus all of these properties influence the resultant photocatalytic activity.

Nowadays, an improvement of photocatalytic activity mainly focuses on more efficient light harvesting, which means the ability of use of overall solar spectrum (a vast range of photoabsorption). To achieve this goal, modification of titania with gold deposits possessing various sizes and shapes is proposed. The first report showing that higher activity was obtained from broader absorption range was presented for large particles of rutile titania modified with both gold NPs and nanorods (NRs) of various sizes [59]. In those samples, LSPR possessed two main absorption peaks at shorter (ca. 520 nm) and longer (ca. 600 nm) wavelengths, due to the presence of gold NRs (transverse and longitudinal LSPR). Since then, gold NRs have been extensively studied for plasmon-assisted photocatalysis, e.g., gold NR as a core for trilayered Au/Ag/TiO_2_ [80] and gold NRs with different aspect ratio [81], [82]. NRs are more efficient nanostructures than NPs, mainly because of their ability of absorbing light in a broader wavelength range, i.e., 400–1000 nm for NRs against 400–700 nm for NPs [83]. Interestingly, Au/OAPs samples possess slightly higher photocatalytic activity than that of the most active commercial titania photocatalysts with very broad LSPR (containing both gold NPs and NRs), and ca. six-fold higher activity than that of fine anatase titania (ST01 from Ishihara) with fine gold NPs similar to that in Au/OAPs samples, as shown in Fig. 8. It is thought that intrinsic properties of OAPs, i.e., fast transfer of photogenerated electrons (here plasmonic electrons) due to preferential distribution of shallow than deep ETs in OAPs [11], are responsible for very high photocatalytic activity of Au/OAPs. To improve the photocatalytic performance of plasmonic photocatalysts, various modifications of morphology are proposed, mainly to hinder back electron transfer from conduction band of titania to gold NPs (Au → TiO_2_ → Au). For example, an improved structure of support was proposed by Z. Bian et al., i.e., “advanced superstructure system” [84]. This structure is based on titania mesocrystals in which electrons from gold NPs migrated through the titania nanocrystal networks from the basal surfaces to the edges of the plate-like mesocrystals, where they are temporary stored for further reactions. Due to this anisotropic electron flow, which hinders recombination of electrons and holes in the gold NPs, this structure results in longer electron lifetime, and therefore enhanced photocatalytic activity by more than one order of magnitude, as compared to that of conventional particulate Au/TiO_2_ system. Therefore, it is proposed that fast electron transfer inside faceted anatase (similar to that in the superstructure system) is responsible for enhanced photocatalytic activity of Au/OAPs.

It must be pointed that deposition of gold on OAPs samples resulted in the highest photocatalytic activity for the 1US/4.5HT sample, under both UV/vis and vis irradiation, as shown for samples prepared with different HT durations (Fig. 7(right)). Interestingly, the best photocatalytic performance was not obtained for Au/OAPs sample having the best titania morphology (1US/6HT, and the best photocatalytic activity among all unmodified OAPs samples [11]), and thus the properties of gold NPs rather than titania morphology seemed to be decisive for photocatalytic activity under visible light irradiation. However, it was impossible to distinguish the impact of the titania morphology from the influence of the properties of gold deposits. In this regard, the OAPs sample having the best morphology (prepared with 1-h US and 6-h HT) was modified with gold by different methods, and obtained results are discussed in the following section.

### 3.2 Size-controlled gold NPs on OAPs with the same physicochemical and surface properties

#### 3.2.1 Preparation and characterization of Au/OAPs

**Fig. 9:**
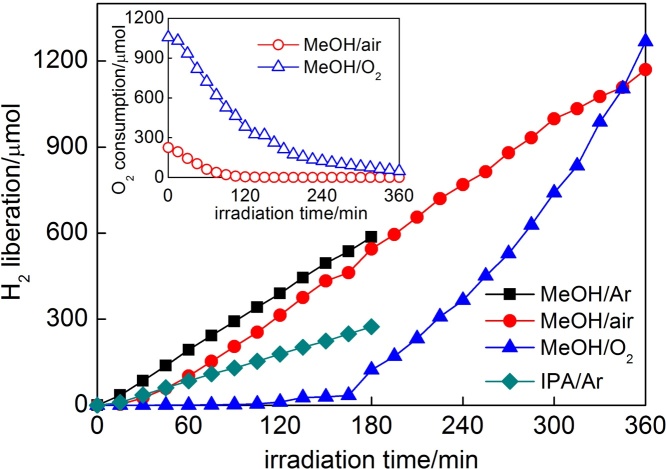
Hydrogen evolution and disappearance of oxygen (inset) during gold photodeposition on OAPs prepared for 1-h US and 6-h HT.

In–situ photodeposition of gold on OAPs caused dehydrogenation of alcohols, and the fastest hydrogen evolution (almost without induction period) was observed for methanol as a hole scavenger under Ar atmosphere (MeOH/Ar), as shown in Fig. 9. In the presence of O_2_ in the system (MeOH/air, MeOH/O_2_), the induction period (ca. 15 min and 60 min, respectively), during which photogenerated electrons were mainly consumed by oxygen (inset in Fig. 9) instead of reducing gold(III), was observed. The change of hole scavenger from MeOH to IPA resulted in significant decrease in efficiency of hydrogen evolution, possibly due to lower polarity of IPA than that of MeOH. It was reported that efficiency of hydrogen evolution was highly dependent on properties of hole scavenger such as polarity, the number of hydroxyl groups, the number of alpha-hydrogens and standard oxidation potential [85].

Au-OAPs samples exhibited dark violet color similar to the data shown in the previous section, indicating the presence of spherical gold NPs of few nms [59]. Diffuse reflectance spectra of all samples did not differ significantly with λ_max_ of LSPR at ca. 545 nm (Fig. S3), and slight stronger scattering at longer wavelengths (800–1200 nm) for samples prepared under Ar suggesting the presence of gold NPs with larger sizes in those samples [63].

**Fig. 10:**
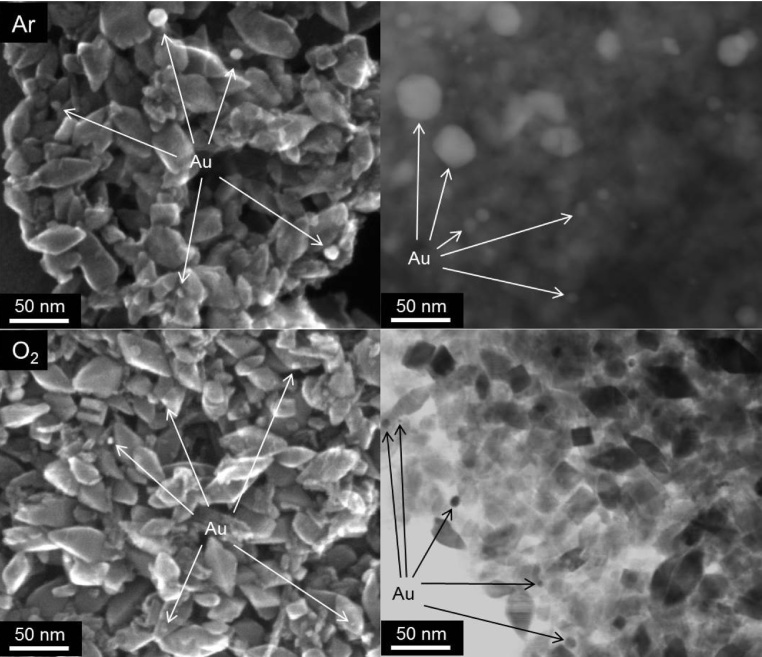
STEM images of Au/OAPs samples: MeOH/Ar (top) and MeOH/O_2_ (bottom).

**Fig. 11:**
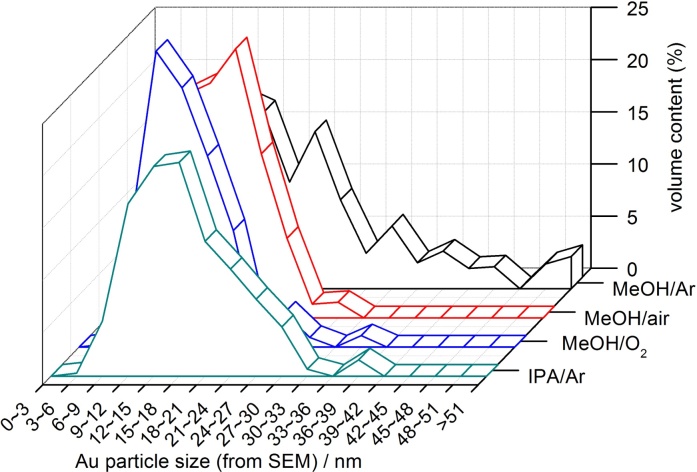
Distribution of gold NPs’ size for Au/OAPs samples prepared under different conditions of gold photodeposition.

It must be pointed that not only average (or mean) size of gold NPs, but size distribution is crucial, especially for visible light activity. Previous study suggested that the highest activity of Au/TiO_2_ samples was caused by broad LSPR peak (greyish sample) of gold deposits possessing various sizes and shapes (spherical and rod-like; with high photocatalytic activity shown in Fig. 8 (top right)) [59]. In those samples, the size of gold NPs directly correlated with the size of titania particles, similarly as in the present study where the change in US-HT conditions has resulted in the change in titania morphology and thus simultaneously influencing the properties of gold deposits. Therefore, single dependence between the size of gold NPs and photocatalytic activity could not be obtained. In addition, it should be pointed that even reports showing the dependence of photocatalytic activity on properties of gold NPs are not unequivocal since various surfactants [70], capping [86] and stabilizing [75] agents, which are used for controlling the size and the shape of gold NPs, can simultaneously influence the resultant overall photocatalytic activity. Here, by modification of only photodeposition conditions of gold NPs on the same titania sample (OAPs prepared at the same conditions of US-HT), it was possible to obtain the impurity-free products (without surfactants and capping/stabilizing agents of gold NPs), which differed by only properties of gold NPs (Fig. 10, Fig. 11). As expected, gold NPs were the most polydispersed in Au/OAPs sample prepared under Ar in the presence of MeOH as a hole scavenger (MeOH/Ar), due to very fast gold(III) reduction and formation of gold NPs (as show in Fig. 9). The presence of larger molecule as a hole scavenger (IPA instead of MeOH, IPA/Ar) or O_2_ as an electron scavenger (MeOH/air and MeOH/O_2_) retarded the gold growth significantly. The content of large Au NPs (>30 nm) in the final product decreased in the order: MeOH/Ar (30–80 nm) > IPA/Ar (36–39 nm) > MeOH/O_2_ (33–36 nm) ≥ MeOH/air (27–30 nm).

#### 3.2.2 Photocatalytic activity of Au/OAPs

**Fig. 12:**
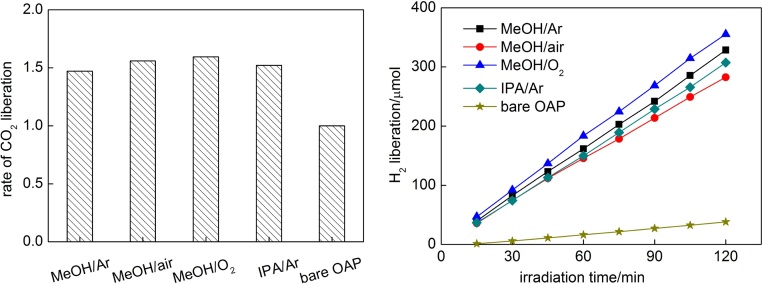
Photocatalytic activity under UV/vis irradiation for Au/OAPs samples for (left) oxidative decomposition of acetic acid, and (right) MeOH dehydrogenation under anaerobic conditions.

Photocatalytic activity under UV/vis irradiation was tested for (1) oxidative decomposition of acetic acid (CO_2_ system), and (2) MeOH dehydrogenation under deaerated conditions (15-min Ar prebubbling (H_2_ system)). Modification of OAPs with gold NPs resulted in significant enhancement of photocatalytic activity (as already reported [59]), being ca. 1.5–1.6 (CO_2_ system) and 6.6-8.3 (H_2_ system) times higher than that of bare OAPs (Fig. 12). Interestingly, similar photocatalytic activity of Au/OAPs samples tested in the presence of the same hole scavenger (MeOH (H_2_ system); 2.57 and 2.94 μmol min^−1^ for samples prepared in the presence of IPA (IPA/Ar) and MeOH (MeOH/Ar), respectively, Fig. 12 (right)) confirms that the kind of a hole scavenger is decisive for efficient hydrogen evolution (as compared with more than twice faster hydrogen evolution from MeOH than IPA suspension during gold photodeposition, as shown in Fig. 9).

Photocatalytic activity of gold-OAP samples under visible light irradiation (λ > 420 nm) was tested for oxidation of IPA. The correlation between the size of gold NPs (both crystallite size estimated by XRD and the size of large gold NPs estimated by SEM) and the photocatalytic activity clarifies that the size of gold NPs is decisive for visible-light activity (MeOH/Ar > IPA/Ar > MeOH/O_2_ > MeOH/air). It should be reminded that for samples containing also nano-sized gold deposits catalytic (dark) activity of gold could not be neglected [59]. Indeed, all Au/OAPs samples exhibited also dark activities, but their levels were much lower than that under irradiation, reaching ca. 22%, 19%, 13% and 18% of the overall activity for MeOH/Ar, MeOH/air, MeOH/O_2_ and IPA/Ar samples, respectively. Interestingly, higher content of dark activity in the MeOH/Ar sample confirmed high polydispersity of gold deposits in this the most active sample (from nano-sized clusters to large NPs of ca. 75 nm).

**Fig. 13:**
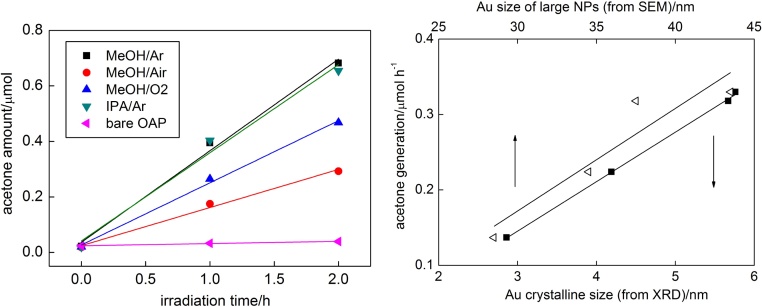
(left) Photocatalytic activity under visible light irradiation for bare and gold-modified OAPs samples, and (right) the correlation between photocatalytic activity and gold size (crystallite and particle).

It must be pointed that differences in UV–vis-activity of those Au/OAPs samples in both tested systems (CO_2_ and H_2_) are not large (<8% and <20%, respectively, as shown in Fig. 12), indicating that size and distribution of gold NPs is not decisive for UV/vis-photocatalytic activity, where primary function of gold is mainly to inhibit the recombination of charge carriers (but not to activate titania as it is under vis excitation of LSPR). Larger differences in photocatalytic activity between modified samples in H_2_ system than in CO_2_ system originate from an additional function of gold deposits in the latter system, i.e., as cocatalyst on which hydrogen evolution takes place. The highest UV-activity is observed for the smallest gold NPs, and thus more uniformly distributed on OAPs (less content of bare titania of neglected activity in the final product). The significant difference between photocatalytic activity of Au/OAPs samples is observed only under vis irradiation (Fig. 13) confirming that the size of gold NPs or/and their distribution are decisive for vis activity.

#### 3.2.3 The numerical simulation of light intensity distribution for Au/OAPs

**Fig. 14:**
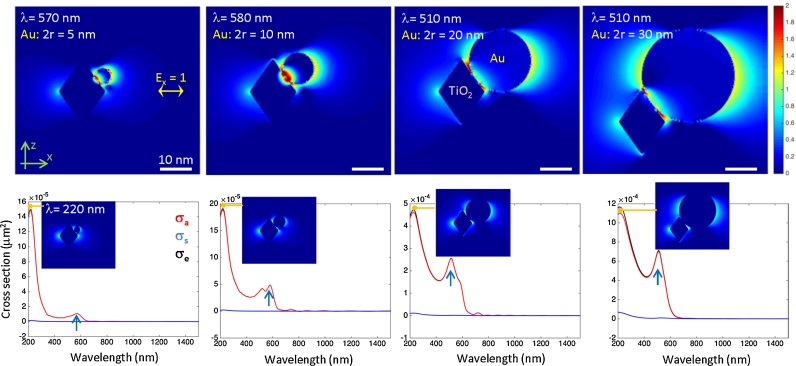
Simulated light intensity enhancement for the different size gold NPs on titania nanocrystal (top row) at the wavelength, λ, corresponding to the plasmon resonance in the extinction spectrum (marked by arrows in the bottom row). Extinction cross section σ_e_ = σ_a_ + σ_s_ is defined by the corresponding absorption, σ_a_, and scattering, σ_s_, cross sections; note difference in the ordinate scales of the plots. The intensity color map is logarithmic and shows an up to a 10^2^ times enhancement localized mainly at the interface between gold and titania. Insets in the extinction spectra show the intensity enhancement at the UV extinction peak around 220 nm. Polarisation of the incident light is linear and E-field is E_x_ = 1. All color maps share the same scale. Gold and titania properties were taken from tabulated databasis (Lumerical).

To clarify the key-factor of vis-activity (size or distribution of gold NPs) the numerical simulation of light intensity distribution for OAPs with different sizes of gold nanosphere attached to it in water was carried out, as shown in Fig. 14. The 3D-FDTD simulations were performed by running the Lumerical FDTD software on a 64-core supercomputing cluster. The domain was cubic with a side length of 60 nm, surrounded by perfectly matched layers to avoid boundary reflections. Realistic sizes and gold NP placements were taken from SEM images, in fact the gold sphere was placed near the central side of the octahedron, so that the contact zone varies in size between 5 and 10 nm according to the sphere diameter, as can be seen in the intensity maps in Fig. 14. This is to better model the real conditions, where the sphere slightly flattens at the contact point, and also to avoid introducing non-physical effects: if the two geometries were touching, the size of the contact point would be the length of a single FDTD mesh element, in this case 0.25 nm, which could bring numerical stability issues to the simulation. A total-field scattered-field (TFSF) method was used to calculate light-field distributions and extinction spectra (total losses due to absorption and scattering). To reveal generic spectral features and field enhancement the modeling was carried out in air and in water, and slight changes occurred due to large refractive index contrast with metal and titania. There were no qualitative differences in light-field enhancement for different locations of gold NP on titania. Optical properties of titania and gold were taken from database and realistically describe optical response. The extinction maxima at 510–570-nm range corresponds to LSPR of gold. The strong extinction at the UV range is due to intraband transitions in gold (from the 5d band to the 6sp band). Recently, it has been reported that this UV excitation of gold in air results in formation of superoxide anion radical (O_2_^•−^), which further reacts with hydroxyl groups at the titania surface to give hydroperoxy radical (HO_2_^•−^) [49]. Titania polymorphs have high real part of refractive index at visible wavelengths, 2.4-2.7, and this helps to create light localization (hot spots) at the interface region between titania and gold NPs (Fig. 14). Up to 100 times of light-field enhancement was observed in the narrow tapering regions close to the interface. For larger gold NPs there was larger light-field enhancement and it was located along the x-direction equatorial cross section of nano-spheres (this is for a x-linearly polarized light).

Coupling between gold and titania causes spectral broadening and some splitting of the resonance peak. This can be a factor for better photocatalytic performance of the titania-gold ensemble. Unfortunately, such splitting was not detected in any measured diffuse reflectance spectra of gold-titania samples (present (Fig. S3) and previous ([59], [70]) studies). It must be clarified that diffuse reflectance spectra are not taken for single gold-titania particle, but for large amount of powdered sample after its pressing, which results in co-interactions between all NPs (e.g., our simulations for titania-gold-titania samples indicates strong scattering, and spectra similar to real diffuse reflectance spectra were obtained (unpublished data)). On the other hand, observed absorption at 400–500 nm and the photocatalytic activity at this region [34] indicate, besides recently reported the d-sp transition (410–440 nm) within gold NPs [75], the coupling between titania and gold as suggested from present simulation. Absorption of titania at wavelengths shorter than 400 nm (E_g_ = 2.9-3.1 eV, depending on polymorphic form) is another factor which makes titania surface charged, and interaction with gold NP can be enhanced due to electronegativity of gold which creates surface charge redistributions. The insets in the extinction spectra plots in Fig. 14 show that enhancement at the 220 nm UV peak of extinction does not cause strong light-field enhancements. At those wavelengths, titania is an absorbing material and its surface is open for surface chemical reactions with gold being an electron reservoir. The 220-nm absorption due to intraband transition in gold has a fast relaxation and this can be the reason for lower than expected activity of gold at UV range. Although an enhancement at short wavelengths is not strong, the surface area over which it is happening, scales with NP size similarly as in the case of plasmonic resonance.

Since the particle sizes are very small, the extinction (total losses) is dominated by absorption rather than by scattering. Such trend is expected to continue up to radius *r* = 30 nm for extinction spectrally integrated over UV-IR spectral range [87]. Absorbed light creates a local heating. This might be a factor which helps for kinetics of chemical reactions via enhanced diffusion, or by molecule activation (plasmonic heating [37], [88]). Although plasmonic heating has recently been rejected as the main mechanism of plasmonic photocatalysis for various reactions (e.g., due to insufficient energy) [36], [89], [90], [91], its participation in overall activity could not be neglected, especially for temperature-sensitive systems, e.g., microorganism deactivation. The local heating contributes in a way similar to an increased surface area over which the light-field enhancement is distributed. This trend of better photocatalytic activity for larger NPs has been observed in experiments and qualitatively supported by this simple modeling using finite exact solver for nanoscale light distribution (Lumerical). It has already been demonstrated that an enhancement of polymerization on arrays of gold nanoparticles takes place at the hot spots and its localization can be controlled by choice of orientation of linear polarisation [92]. Recently, it has been shown that by co-sputtering of different plasmonic metals (copper, silver, and gold), it is possible to tailor optical response of the alloys [93]. We could foresee an possibility for creation of tailored photo-catalysts using such alloys which are created by intermixing of metals in atomic scale. However, it is also important to remember that the field enhancement does not unequivocally indicate the enhanced photocatalytic activity. For example, gold (core)-silver (shell)/titania with strong enhancement of plasmonic field at the interface between core-shell and titania exhibited lower photocatalytic activity than that of titania modified with monometallic NPs, which suggests that second metal works as electron-hole recombination center, and indirectly confirms the mechanism of electron rather than energy transfer for plasmonic degradation of organic compounds under visible-light irradiation [61]. It is also important that the nano-/micrometer-scale surface covered with titania needles creates a gradient refractive index, which acts as antireflective broad-band coating similar to the textures of some natural surfaces [94]. Such broad-band absorption can further contribute to efficiency of photocatalysts by both the most probable mechanisms, i.e., energy and electron transfer.

## 4 Summary and conclusions

OAPs were prepared by a US-HT method under varied preparation conditions (HT duration and US duration). Generally, gold NPs were photodeposited on OAPs under Ar (to avoid electron scavenging by O_2_) and in the presence of MeOH working as a hole scavenger (to avoid hole recombination with electrons). It was found that morphology of titania governed properties of gold deposits, i.e., the better the morphology was (the larger content of faceted titania, OAPs), the larger were gold NPs. This finding suggests that surface defects work as active sites for nucleation and for deposition of gold NPs.

Titania modification with gold NPs significantly enhanced photocatalytic activities under UV/vis in both tested systems (aerobic decomposition of acetic acid and anaerobic dehydrogenation of MeOH) and activated titania towards visible light. Enhancement of activity has already been reported and is caused by: (i) inhibition of electron-hole recombination since noble-metal NPs work as an electron sink, and (ii) titania sensitization by LSPR of gold NPs, under UV and vis light, respectively. Although our previous study clearly proved that OAP morphology governs photocatalytic activity, due to preferential distribution of shallow than deep ETs [11], the highest photocatalytic activity of OAPs modified with gold NPs was achieved not for the gold-OAP sample of the best morphology (prepared for 1-h US and 6-h HT). This finding suggests that properties of gold NPs are decisive for resultant photocatalytic activity.

The change of US-HT conditions resulted in the change of all surface properties of titania as well as its morphology (content of OAPs), and thus simultaneously influenced the properties of gold NPs. Therefore single correlation between gold properties and resultant photocatalytic activity could not be drawn. In this regard, new methods of gold photodeposition, varying with the kind of a hole scavenger (MeOH, IPA) and an electron scavenger (air, O_2_, Ar-with no scavenging abilities), was applied. Under UV/vis irradiation the change in gold size resulted in slight change in photocatalytic activity, i.e., the highest activity was obtained for the MeOH/O_2_ sample with the smallest sizes of gold NPs, and thus more uniformly distributed on OAPs. On the contrary, under vis irradiation significant enhancement of activity was observed for MeOH/Ar sample with the largest and most polydispersed gold NPs. Although clear correlation between the size of gold NPs and photocatalytic activity suggests that the size of gold NP is a key-factor of vis-activity, the polydispersity of gold deposits, and thus ability of photoabsorption of more photons (broad LSPR), could not be neglected. Therefore, 3D-FDTD simulations were performed to reveal spectral and spatial features of the plasmonic field. They corroborated experimental observations that for larger gold NPs the larger surface areas have field enhancement.
