# Supplementary material for: Size-controlled gold nanoparticles on octahedral anatase particles as efficient plasmonic photocatalyst
Source: Appl Catal B. 2017 Jun 5;206:393–405. doi: 10.1016/j.apcatb.2017.01.043 (PMC5327951; doi:10.1016/j.apcatb.2017.01.043)
Supplement: Supplementary file 1 [file mmc1.pdf]

Electronic supplementary information for

Size-controlled gold nanoparticles on octahedral anatase particles as efficient plasmonic photocatalyst

Zhishun Wei,[1] Lorenzo Rosa,[2,3] Kunlei Wang,[1] Maya Endo,[1] Saulius Juodkazis,[2] Bunsho Ohtani,[1] and Ewa Kowalska[1,*]

[1]Institute for Catalysis, Hokkaido University, N21 W10, 001-0021 Sapporo, Japan ([*] kowalska@cat.hokudai.ac.jp)
[2]Centre for Micro-Photonics, Swinburne University of Technology, PO Box 218, Hawthorn, 3122 Australia
[3]Department of Information Engineering, University of Parma, V.le G.P. Usberti 181/A, I-43124 Parma, Italy

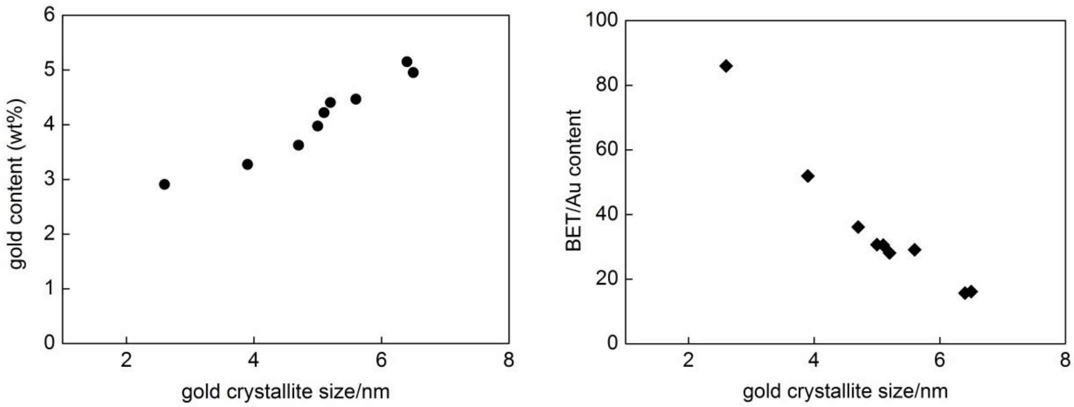

Fig. S1. (left) The correlation between crystallite size of gold NPs (estimated from XRD) and surface content of gold (wt%, estimated by XPS), and (right) The correlation between the crystallite size of gold NPs (estimated from XRD) and the ratio of specific surface area to surface content of gold.

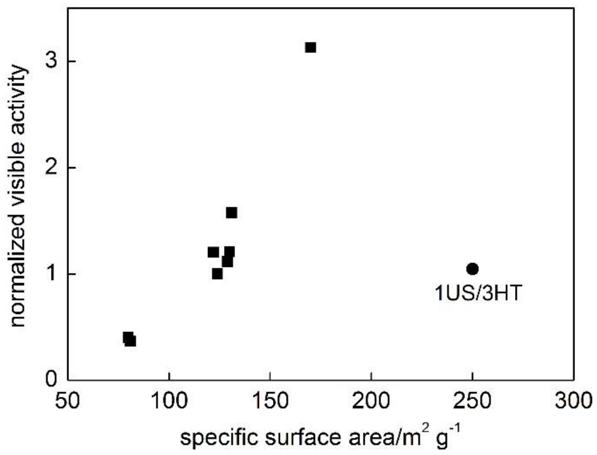

Fig. S2. The correlation between specific surface area and vis activity for Au/OAPs samples (1US/3HT in red)

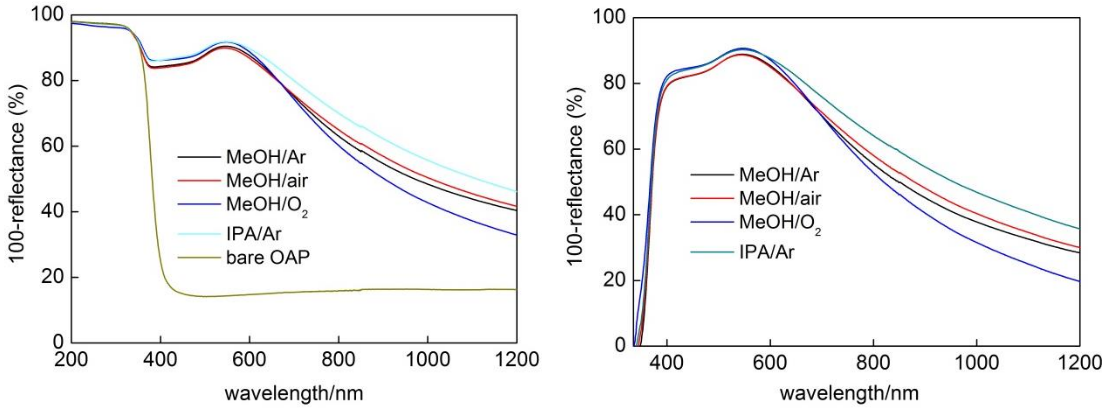

Fig. S3. Diffuse reflectance spectra of Au/OAP (DRS colors correspond to the Fig. 11).
